# Draft Genome Sequence of *Pseudomonas fluorescens* Strain TR3, a Potential Biocontrol Agent against the Rice Blast Fungus *Magnaporthe oryzae*

**DOI:** 10.1128/genomeA.01332-17

**Published:** 2017-11-22

**Authors:** Xue Zhang, Jin-Rong Xu, Cindy H. Nakatsu

**Affiliations:** a Department of Botany and Plant Pathology, Purdue University, West Lafayette, Indiana, USA; b Department of Agronomy, Purdue University, West Lafayette, Indiana, USA

## Abstract

We present the draft genome sequence of the potential biocontrol agent *Pseudomonas fluorescens* TR3, which was isolated from rice leaves infected with *Magnaporthe oryzae* in a greenhouse. The genome of TR3 was assembled into 26 scaffolds (~6 Mbp) and includes genes potentially involved in bacterial interactions with fungi.

## GENOME ANNOUNCEMENT

*Pseudomonas fluorescens* is a Gram-negative rod-shaped bacterium that commonly inhabits water, soil, and plant rhizospheres. *P. fluorescens* strains are of interest to agricultural and environmental scientists because of their ability to produce a variety of antimicrobial secondary metabolites to suppress pathogens (1). Currently, the whole genomes of several *P. fluorescens* strains that exhibit antifungal activity, such as *P. fluorescens* strains Pf-5, CHA0, F113, SS101, KD, and LBUM223 (2–5), have been sequenced. *P. fluorescens* strains Pf-5, CHA0, and F113 produce a spectrum of antibiotics, such as 2,4-diacetylphloroglucinol (DAPG) (4, 6, 7), hydrogen cyanide (HCN) (8), and pyoluteorin (2, 9), which are active against various phytopathogenic fungi (10). The type III secretion system (T3SS) is involved in inhibition of *Pythium ultimum* by strain KD (3). To date, no strain of this species has been tested for antifungal activity against *Magnaporthe oryzae*.

The genomic DNA was isolated according to a basic phenol-chloroform purification protocol for *Pseudomonas* spp. (11). Whole-genome shotgun sequencing of *P. fluorescens* strain TR3 was performed by using a HiSeq 2500 (Illumina, Inc., San Diego, CA, USA) instrument and a 100-bp paired-end library (DNA PCR-free sample prep kit [Illumina]), Genomic contigs were *de novo* assembled using ABySS (12). A total of 42,852,340 reads (4.1 × 10^9^ total bases) were assembled *de novo* into 26 scaffolds totaling 6,158,893 bp with a G+C content of 59.9%. To order the contigs, the *P. fluorescens* strain TR3 draft genome was matched to a reference genome, *P. fluorescens* strain Pf0-1 (GenBank accession number NC_007492). We predicted 5,625 genes with Rapid Annotation using Subsystem Technology (RAST) (13) and COG (14) in the NCBI Prokaryotic Genome Annotation Pipeline (PGAP) tools, 12 rRNA operons with RNAmmer (15), and 44 tRNA genes with tRNAscan-SE (16).

Multilocus sequence-based phylogenetic analysis (MLSA) was performed using three housekeeping genes, *gyrB* (2,418 bp), *rpoD* (1,848 bp), and 16S rRNA (1,139 bp). There was >98% sequence similarity to *P. fluorescens* A506 (17), confirming the phylogenetic position of the species. Additional phylogenetic analysis was performed using whole-genome-based average nucleotide identity (ANI) analysis (18, 19). The ANI calculator computed 95.70% ANI shared between strain TR3 and *P. fluorescens* A506.

*P. fluorescens* TR3 was isolated from rice leaves infected with the fungal plant pathogen *Magnaporthe oryzae* in the greenhouse. Strain TR3 inhibits fungal growth in a contact-based manner. Genome sequence analysis revealed various genes that code for proteins potentially related to bacterial and fungal interaction, including gene clusters for antibiotics (e.g., pyoverdine phenazine), chitinase, metabolites for plant-bacterium communications (e.g., catabolism of acetoin and 2,3-butanediol), and potential mechanisms for bacterial and fungal contact, including T3SS and the type II (T2SS), type IV (T4SS), and type VI (T6SS) secretion systems and the widespread colonizing island (WCI) (20). The draft genome sequence of *P. fluorescens strain* TR3 presented here is a source of information for bacterial and fungal interactions.

### Accession number(s).

The genome sequence of strain TR3 was deposited in GenBank under the accession number MKHA00000000. The version described in this paper is the first version.

## References

[1] O’Sullivan DJ, O’Gara F 1992 Traits of fluorescent *Pseudomonas* spp. involved in suppression of plant root pathogens. Microbiol Rev 56:662–676. 1480114 10.1128/mr.56.4.662-676.1992 PMC372893

[2] Ramette A, Frapolli M, Fischer-Le Saux M, Gruffaz C, Meyer JM, Défago G, Sutra L, Moënne-Loccoz Y 2011 *Pseudomonas protegens* sp. nov., widespread plant-protecting bacteria producing the biocontrol compounds 2,4-diacetylphloroglucinol and pyoluteorin. Syst Appl Microbiol 34:180–188. doi:10.1016/j.syapm.2010.10.005. 21392918

[3] Rezzonico F, Binder C, Défago G, Moënne-Loccoz Y 2005 The type III secretion system of biocontrol *Pseudomonas fluorescens* KD targets the phytopathogenic *Chromista Pythium ultimum* and promotes cucumber protection. Mol Plant Microbe Interact 18:991–1001. doi:10.1094/MPMI-18-0991. 16167769

[4] Howell CR, Stipanovic RD 1980 Suppression of *Pythium ultimum* induced damping-off of cotton seedlings by *Pseudomonas fluorescens* and its antibiotic, pyoluteorin. Phytopathology 70:712–715. doi:10.1094/Phyto-70-712.

[5] Woese CR, Kandler O, Wheelis ML 1990 Towards a natural system of organisms: proposal for the domains *Archaea*, *Bacteria*, and *Eucarya*. Proc Natl Acad Sci U S A 87:4576–4579. doi:10.1073/pnas.87.12.4576. 2112744 PMC54159

[6] Howell CR, Stipanovic RD 1979 Control of *Rhizoctonia solani* on cotton seedlings with *Pseudomonas fluorescens* and with an antibiotic produced by the bacterium. Phytopathology 69:480–482. doi:10.1094/Phyto-69-480.

[7] Loper JE, Kobayashi DY, Paulsen IT 2007 The genomic sequence of *Pseudomonas fluorescens* Pf-5: insights into biological control. Phytopathology 97:233–238. doi:10.1094/PHYTO-97-2-0233. 18944380

[8] Redondo-Nieto M, Barret M, Morrisey JP, Germaine K, Martínez-Granero F, Barahona E, Navazo A, Sánchez-Contreras M, Moynihan JA, Giddens SR, Coppoolse ER, Muriel C, Stiekema WJ, Rainey PB, Dowling D, O’Gara F, Martín M, Rivilla R 2012 Genome sequence of the biocontrol strain *Pseudomonas fluorescens* F113. J Bacteriol 194:1273–1274. doi:10.1128/JB.06601-11. 22328765 PMC3294817

[9] Paulsen IT, Press CM, Ravel J, Kobayashi DY, Myers GSA, Mavrodi DV, DeBoy RT, Seshadri R, Ren QH, Madupu R, Dodson RJ, Durkin AS, Brinkac LM, Daugherty SC, Sullivan SA, Rosovitz MJ, Gwinn ML, Zhou LW, Schneider DJ, Cartinhour SW, Nelson WC, Weidman J, Watkins K, Tran K, Khouri H, Pierson EA, Pierson LS, Thomashow LS, Loper JE 2005 Complete genome sequence of the plant commensal *Pseudomonas fluorescens* Pf-5. Nat Biotechnol 23:873–878. doi:10.1038/nbt1110. 15980861 PMC7416659

[10] Majumder D, Kongbrailatpam JD, Suting EG, Kangjam B, Lyngdoh D 2014 *Pseudomonas fluorescens*: a potential biocontrol agent for management of fungal diseases of crop plants, p 317–342. *In* Goyal A, Manoharachary C (ed), Future challenges in crop protection against fungal pathogens. Springer, New York, NY. doi:10.1007/978-1-4939-1188-2_11.

[11] Clarke L, Millar BC, Moore JE 2003 Extraction of genomic DNA from *Pseudomonas aeruginosa*: a comparison of three methods. Br J Biomed Sci 60:34–35. 12680631 10.1080/09674845.2003.11978040

[12] Simpson JT, Wong K, Jackman SD, Schein JE, Jones SJ, Birol I 2009 ABySS: a parallel assembler for short read sequence data. Genome Res 19:1117–1123. doi:10.1101/gr.089532.108. 19251739 PMC2694472

[13] Aziz RK, Bartels D, Best AA, DeJongh M, Disz T, Edwards RA, Formsma K, Gerdes S, Glass EM, Kubal M, Meyer F, Olsen GJ, Olson R, Osterman AL, Overbeek RA, McNeil LK, Paarmann D, Paczian T, Parrello B, Pusch GD, Reich C, Stevens R, Vassieva O, Vonstein V, Wilke A, Zagnitko O 2008 The RAST server: Rapid Annotations using Subsystems Technology. BMC Genomics 9:75. doi:10.1186/1471-2164-9-75. 18261238 PMC2265698

[14] Wu S, Zhu Z, Fu L, Niu B, Li W 2011 WebMGA: a customizable Web server for fast metagenomic sequence analysis. BMC Genomics 12:444. doi:10.1186/1471-2164-12-444. 21899761 PMC3180703

[15] Lagesen K, Hallin P, Rødland EA, Staerfeldt HH, Rognes T, Ussery DW 2007 RNAmmer: consistent and rapid annotation of ribosomal RNA genes. Nucleic Acids Res 35:3100–3108. doi:10.1093/nar/gkm160. 17452365 PMC1888812

[16] Lowe TM, Eddy SR 1997 TRNAscan-SE: a program for improved detection of transfer RNA genes in genomic sequence. Nucleic Acids Res 25:955–964. 9023104 10.1093/nar/25.5.955 PMC146525

[17] Stockwell VO, Johnson KB, Sugar D, Loper JE 2010 Control of fire blight by *Pseudomonas fluorescens* A506 and *Pantoea vagans* C9-1 applied as single strain and mixed inocula. Phytopathology 100:1330–1339. doi:10.1094/PHYTO-03-10-0097. 20839963

[18] Varghese NJ, Mukherjee S, Ivanova N, Konstantinidis KT, Mavrommatis K, Kyrpides NC, Pati A 2015 Microbial species delineation using whole-genome sequences. Nucleic Acids Res 43:6761–6771. doi:10.1093/nar/gkv657. 26150420 PMC4538840

[19] Goris J, Konstantinidis KT, Klappenbach JA, Coenye T, Vandamme P, Tiedje JM 2007 DNA-DNA hybridization values and their relationship to whole-genome sequence similarities. Int J Syst Evol Microbiol 57:81–91. doi:10.1099/ijs.0.64483-0. 17220447

[20] Scales BS, Erb-Downward JR, Huffnagle IM, LiPuma JJ, Huffnagle GB 2015 Comparative genomics of *Pseudomonas fluorescens* subclade III strains from human lungs. BMC Genomics 16:1032. doi:10.1186/s12864-015-2261-2. 26644001 PMC4672498

